# Epidemiological characteristics and risk distribution prediction of severe fever with thrombocytopenia syndrome in Zhejiang Province, China

**DOI:** 10.1371/journal.pntd.0013066

**Published:** 2025-04-25

**Authors:** Rongrong Qu, Ying Liu, Mengsha Chen, Huihui Zhang, Qianqian Feng, Shigui Yang, Jimin Sun

**Affiliations:** 1 Department of Emergency Medicine, Second Affiliated Hospital, Department of Epidemiology and Biostatistics, School of Public Health, The Key Laboratory of Intelligent Preventive Medicine of Zhejiang Province, Zhejiang University School of Medicine, Hangzhou, China; 2 Zhejiang Key Lab of Vaccine, Infectious Disease Prevention and Control, Zhejiang Provincial Center for Disease Control and Prevention, Hangzhou, China; Connecticut Agricultural Experiment Station, UNITED STATES OF AMERICA

## Abstract

**Background:**

Severe fever with thrombocytopenia syndrome (SFTS) is an emerging infectious disease, garnering increasing attention due to rising case numbers and expanding geographical reach. However, there is limited research on the potential factors influencing the distribution of SFTS.

**Methods:**

Data on SFTS cases in Zhejiang province were obtained from 2011 to 2022. Data on ecoclimatic factors, land cover, and human population density at the county level were also collected. Spatial autocorrelation analysis was used to analyze the epidemic characteristics and spatial clustering. A boosted regression tree (BRT) model was used to assess ecoclimatic and socioenvironmental drivers for the distributions of SFTS.

**Results:**

The SFTS cases increased from 9 in 2011–1,103 in 2022 with an average incidence rate of 0.099 per 100,000. There is an obvious seasonality to SFTS cases, primarily occurring between April and August. We detected global spatial autocorrelation of SFTS cases in all years (P < 0.05) except 2011, 2012 and 2014. Local spatial autocorrelation analysis suggested that the “High-high” agglomeration areas are mainly distributed in the hilly terrain of the east coast of Zhejiang province. Furthermore, factors such as mean temperature of wettest quarter (relative contribution, RC = 18.51%) and annual precipitation (9.29%) were found to have significantly contribution to the occurrence of SFTS. The model-predicted risk areas, particularly in Daishan County (predicted probability of cases: 0.986), Linhai city (0.972), and Tiantai County (0.971), align with reported cases.

**Conclusions:**

These findings suggested that SFTS incidence has increased and spatially expanded over the past few years. It is necessary to expand the scope and improve the sensitivity of surveillance, especially in western, southern and northern Zhejiang.

## Summary

Severe fever with thrombocytopenia syndrome (SFTS) has emerged as a significant public health concern in Zhejiang province, with cases rising from 9 in 2011–1,103 in 2022. The incidence exhibited clear seasonality, peaking between April and August. Additionally, the cases also showed spatial clustering, particularly in the hilly regions of eastern Zhejiang. Our study identified key ecoclimatic factors such as mean temperature of the wettest quarter and annual precipitation as significant contributors to SFTS occurrence using boosted regression trees (BRT). The model accurately predicted high-risk areas, including Daishan County, Linhai City, and Tiantai County, aligning with reported cases. These findings underscore the need for enhanced surveillance and preventive measures, particularly in western, southern, and northern Zhejiang, to mitigate the growing threat of SFTS.

## Introduction

Severe fever with thrombocytopenia syndrome (SFTS) is an emerging infectious disease caused by a novel highly lethal bunyavirus called the SFTS virus (SFTSV), which is classified in the order Bunyavirales, family Phenuiviridae, and genus Phlebovirus [1]. This disease was first reported in rural areas of Henan and Hubei provinces in China in 2009, and subsequently found in South Korea and Japan in 2012 and 2013 [2,3]. The average case fatality rate (CFR) of SFTSV infection in China is around 7.3% (2391 cases and 174 deaths), ranging from 6.3% to 30.0% in multiple studies as reported in 2014 [4]. A prospective observational study in Xinyang, China found the CFR of SFTS patients was 16.2% (95% CI 14.6-17.8) [5]. However, researchers have proposed that the case fatality rate of SFTS may be underestimated. These differences may be due to varying detection or reporting rates in different regions.

The common manifestations of SFTSV are symptoms such as fever high fever, gastrointestinal symptoms, thrombocytopenia, leukopenia, and lymphadenopathy and sometimes even multiple organ failure leading to death [6]. Generally, SFTSV is primarily directly transmitted to humans through bites from infected ticks such as Haemaphysalis longicornis (H. longicornis) and Rhipicephalus microplus [7] or through contact with blood or tissues of infected animals such as cattle, goats, sheep, dogs, pigs, and poultry [8,9]. Human-to-human transmission is also a route of transmission as which has observed in a few family and nosocomial clusters of cases. Specifically, several studies have speculated SFTSV is capable of sexual transmission or aerosols transmission through body fluids and secretions [10,11]. Nonetheless, there is currently no definitive evidence for airborne transmission of SFTSV. In addition, climatic factors and environmental factors could directly affect the tick-growth dynamics and virus replication process thus influencing the tick-human interactions [12]. Social factors can also play an indirect role in the spread of the virus, for examine, density of domestic animals [13].

In 2017, SFTS was listed one of the most serious emerging infectious diseases by World Health Organization. Moreover, in 2018 annual review of the Blueprint list of priority diseases, SFTS was also prioritised for research and development [1]. However, there is no effective vaccine available for prevention. The heterogeneity of spatial-temporal transmission of SFTS may be driven by numerous factors. The geographic distribution of SFTS has expanded worldwide in recent years [14], but there is limited research on the potential factors influencing its spread. Therefore, SFTS remains a serious infectious disease and poses an increasing threat to public health and further studies on this aspect are required.

The annual number of cases and affected counties in China also have been rising year by year. There are 90 counties in Zhejiang, and the incidence rate of SFTS ranks fifth in China [15]. Interestingly, the top four provinces including Henan, Shangdong, Hubei, and Anhui are all located in northern or central China, while Zhejiang Province is located the region of southeast China [16]. With this background, the aim of this study was to investigate the epidemiological characteristics of SFTS in Zhejiang from 2011 to 2022. Furthermore, the association of the spatial distribution of SFTS with various factors was explored to find the range of suitable habitats for the transmission of SFTS and to implement control measures at an early time.

## Materials and methods

### Data collection

Zhejiang Province is a densely populated and economically developed province located in the southeastern Yangtze River Delta of China, covering a total area of 101,800 km2, with 11 cities namely Hangzhou, Ningbo, Wenzhou, Jiaxing, Huzhou, Shaoxing, Jinhua, Quzhou, Zhoushan, Taizhou, and Lishui city administratively [17] (S1 Fig). Mountains and hills dominate the terrain, and there is a saying that “seven mountains, one water, and two fields” [18]. The SFTS case definition was based on guideline published by National Health and Family Planning Commission of the People’s Republic of China (http://www.moh.gov.cn/mohwsyjbgs/s8348/201010/49272.shtml. Accessed: 08 Oct 2016). Suspected cases are those with epidemiological history (working, living, or traveling in hilly, forest, mountainous areas during the epidemic season, or a history of tick bites within 2 weeks before onset), fever, and other clinical manifestations, as well as peripheral thrombocytopenia and leukopenia. Confirmed cases are those suspected cases who meet one of the following conditions: (1) positive nucleic acid test for novel Bunyavirus in case specimens; (2) positive conversion of IgG antibody to novel Bunyavirus in case specimens or an increase in titer of more than 4 times higher than the acute phase in case specimens; (3) isolation of novel Bunyavirus in case specimens). Medical personnel are required to report all confirmed cases of SFTS in humans to the China Information Network System for Disease Prevention and Control. The surveillance data of SFTS in Zhejiang Province during 2011–2022 was obtained from Zhejiang Information System for Disease Control and Prevention. The collected information includes age, gender, occupation, residential address, the date of illness onset, the date of diagnosed, and the date of death. In addition, the human population for each county was collected obtained by the year for further analysis from Zhejiang Provincial Bureau of Statistics. The data on land cover from the 30 m annual land cover grid data in China [19] and the 19 cross-sectional ecoclimatic variables (BIO01‒19, also called bioclimatic variables recommended by the U.S. Geological Survey) including annual mean temperature, annual precipitation and other variables were also obtained [20]. According to the ‘Law on Prevention and Control of Infectious Diseases’ of The People’s Republic of China, there is no need for ethical approval and informed consent in case of short investigations on SFTS patients, which are performed by medical institutions. The datasets used are included in the supplementary materials (S1 Data, S2 Data and S3 Data).

### Descriptive analysis and spatial autocorrelation analysis

The case database of SFTS from 2011-2022 in Zhejiang was established using Excel 2010 software. The epidemiological characteristics such as spatial and temporal distribution of SFTS patients were described. The comparison of different rates was conducted using the χ² test. A P value less than 0.05 represented statistical significance for all the tests. Based on the survey data, the spatial autocorrelation of the incidence of SFTS was analyzed carried out by Geoda Software, including global and local spatial autocorrelation statistics [21]. The main purpose of global spatial autocorrelation is to describe the spatial distribution pattern of SFTS in the whole study area, especially whether there is significant spatial clustering. The value of Moran’s I statistics is between [-1, 1]. I > 0 shows that there is a positive spatial correlation between research objects, which means 0 is random distribution, while I < 0 shows negative spatial correlation. The significance of Moran’s I was evaluated by z-test, and the test level was α = 0.05. The z statistics and values are based on the results of the Monte Carlo simulation with the number of replications set to 999.

Local spatial autocorrelation is used to further explore the correlation between the values of the variables in a specific spatial location and the values of the variables in the adjacent and adjacent locations, so to identify the hot spots of the disease. This method usually uses local indicators of spatial association (LISA) to draw an aggregate map, which shows the distribution of local spatial autocorrelation intuitively. The LISA aggregation map is divided into four types: high value and high value (H-H), low value and low value (L-L), high value and low value (H-L), low value and high value (L-H). H-H clusters indicate a high-value region surrounded by other high-value regions, L-L clusters indicate a low-value region surrounded by other low-value regions, a H-L cluster means a high-value area is surrounded by a low-value area, and a L-H cluster means a low-value area is surrounded by a high-value area.

### Ecological modeling

For each SFTS patient, a case-control study design was employed to build predictive machine-learning models at the county level. Briefly, case counties were indeed defined based on the aggregation of cases across the entire study period from 2011 to 2022. A county was classified as a case county if it reported at least one case during the study period. Boosted Regression Trees (BRT) model at the county level was built to the training set to evaluate the relative contributions of ecoclimatic, land cover, and social factors to the geographic distribution of SFTS. The BRT model prevailing in ecological studies, is an ensemble method combining the advantages of two algorithms, regression trees and machine learning techniques, which allows nonlinear associations between outcomes and covariates or multicollinearity between covariates [22]. For this study, variables including 19 ecoclimatic variables, 9 environmental variables and one social variable as potential predictors were selected (S1 and S2 Tables).

In BRT model, tree complexity determines the maximum split depth of each regression tree and thus controls the ability of the model to capture non-linear relationships. We chose a moderate tree complexity. The learning rate controls the weight of each tree’s contribution to the overall model prediction. A lower learning rate usually improves the predictive performance of the model, but more iterations are required to achieve optimal performance. We used a smaller learning rate (0.005). Bagging fraction controls the proportion of training samples used to build the tree in each iteration and we selected a value of less than 1(0.75).

In summary, a tree complexity of 5, a learning rate of 0.005 and a bagging fraction of 75% were used for the primary analysis according to previous research [23]. The output of the BRT model includes both relative contributions of predictors and predicted probabilities of occurrence. In order to choose the optimal number of trees, the gbm.step function in the R dismo package was used. Variables that had a high contribution to the occurrence of SFTS (weight >2%) were included and weight 5% were considered significant.

The following sequential steps were repeated 50 time: first, those counties not reporting SFTS cases were randomly selected and were combined with counties with reporting cases to consist of a balanced bootstrap set (case-control ratio was 1: 5). Second, a training set with 70% of data points was randomly selected and the remaining 30% acted as a test set. Then, the training set was used to construct a BRT model, and then the test set was applied for validation. The ROC curves and areas under the curve (AUC) based on the test sets and train sets were averaged separately to represent the final predictive performance. The ROC curve is a widely utilized metric for assessing the efficacy of a classification model. It measures the capacity of a model to discriminate between classes by depicting how the true positive rate and false positive rate vary with different cutoff points. The Area Under the Curve (AUC) is a measure that reflects the accuracy of a model in distinguishing between positive and negative instances, with values ranging between 0 and 1. Finally, the mean value and standard deviation of relative contributions over 50 resampled datasets were reported. And predicted probabilities were averaged over all models to represent the final estimates of the county-specific probabilities of presence. BRT Modeling was performed using dismo and gbm R packages, and predictive power was evaluated using pROC in the R v4.3.3 environment (https://www.r-project.org).

## Results

### Time distribution of STFS

**Fig 1:**
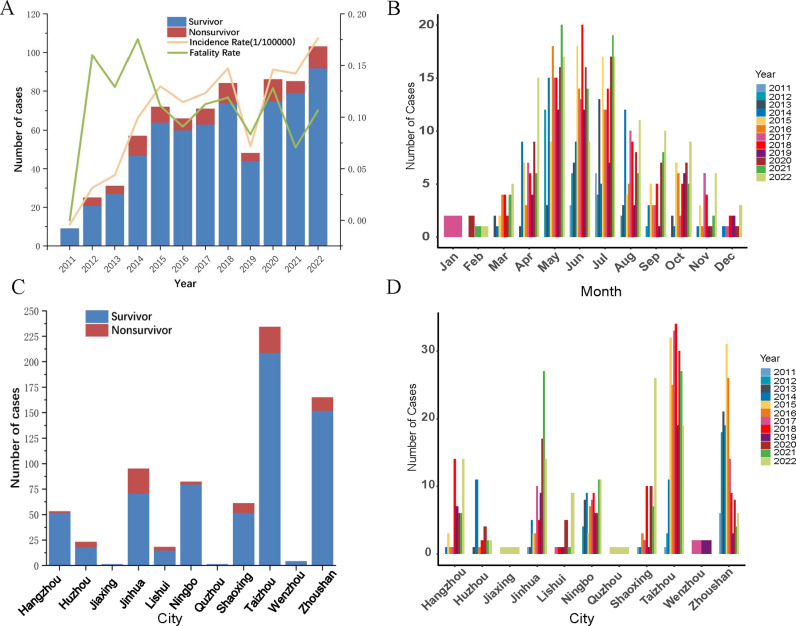
The number of SFTS cases in Zhejiang Province from 2011 to 2022. A, the annual number of SFTS cases, incidence rate, and case fatality rate; B, the monthly case count of SFTS; C, the number of cases of SFTS in various cities; D, the number of cases of SFTS in various cities in different years.

From 2011 to 2022, 737 SFTS cases and 82 deaths were reported in Zhejiang province. The annual number of cases reported showing an increasing trend (Fig 1A). The number of reported deaths was also demonstrated an increased trend. The average annual incidence rate was 0.099/100000. The mortality increased first and then decreased and tended to be stable. The average mortality was 11.1% (82/737), the highest case fatality rate was 17.5% (10/57) in 2014. There was no significant difference in mortality among the years (χ² = 6.592, P = 0.831). Notably, the number of cases at the peak of the disease in 2019 was significantly lower than in other years. The number of months in which cases occurred has been increasing year by year from 2011 to 2022, with only June and July of 2011 occurring the disease. By 2022, there have been cases in all months except for January (Fig 1B). The incidence of SFTS has obvious seasonality, with the onset time concentrated from April to August (S2 Fig). The number of cases starts to increase in March, reaches peak during May to July, and gradually decreases. From April to August in 12 years, Zhejiang Province reported a total of 576 cases, accounting for 78%. The peak period of SFTS mortality is consistent with the peak period of incidence, mainly concentrating from April to August. A total of 67 deaths were reported, accounting for 81.7% (67/82) of the total.

### Spatial distribution of SFTS

**Fig 2:**
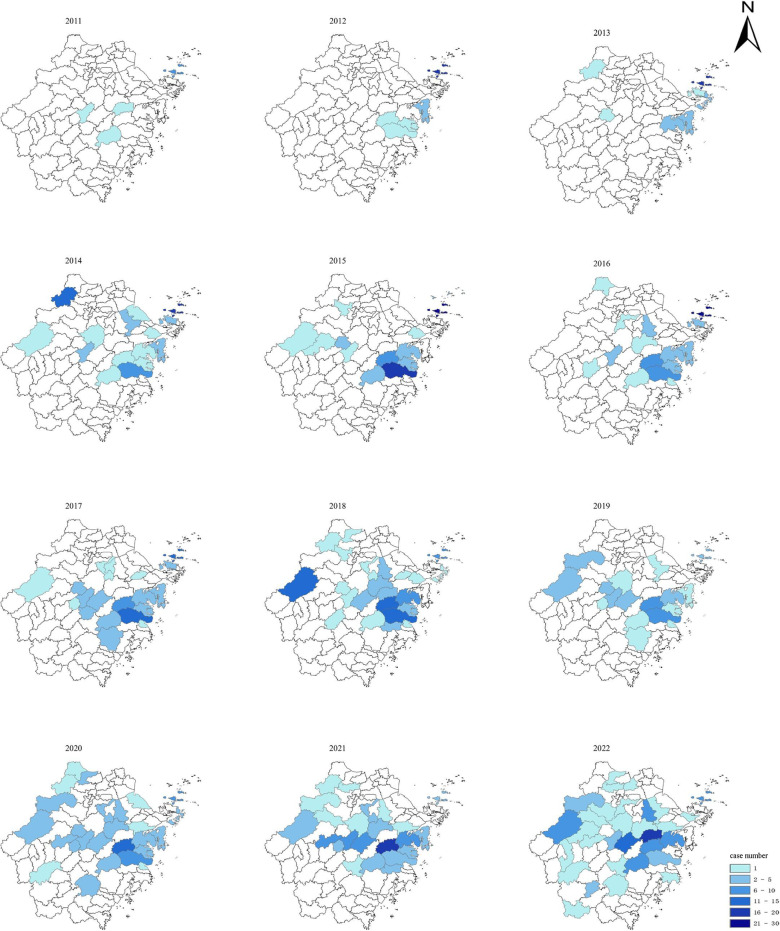
Spatial Distribution Change Map of Cases of SFTS in Zhejiang Province from 2011 to 2022. The base layer of the map is from DataV.GeoAtlas, a website providing standard maps that are approved for public use and are in compliance with the national standards for map representation (http://datav.aliyun.com/portal/school/atlas/area_selector#&lat=31.769817845138945&lng=104.29901249999999&zoom=4).

From 2011 to 2022, SFTS cases were reported in 11 prefectural-level cities of Zhejiang province, among which cumulative cases were reported. The top three prefectural-level cities were Taizhou (234 cases), Zhoushan (165 cases) and Jinhua (95 cases), while the total number of cases in Jiaxing, Quzhou and Wenzhou is only 0.8% (Fig 1C). A total of 56 counties (cities and districts) were reported, and the number of affected counties (cities and districts) increased from 4 in 2011–32 in 2022. The top three cumulative reported cases were in Daishan County (149 cases), Tiantai County (90 cases) and Linhai City (85 cases) (Fig 1D). The distribution of the dead cases was similar with that of the cases. The dead cases were concentrated in Taizhou (25 cases), Jinhua (24 cases) and Zhoushan (13 cases), accounting for 74.7% of the total dead cases (Fig 1C). Notably, about 48.9% (95/194) of SFTS cases occurred in Zhoushan City from 2011 to 2015. However, from 2016 to 2022, about 34.4% (187/543) of cases were concentrated in Taizhou City, and about 15.7% (85/543) of cases were concentrated in Jinhua City. Meanwhile, it was observed that the number of cases in Shaoxing City has sharply increased in 2022, and there is a trend of severe disaster areas shifting to Shaoxing City (Fig 2).

There were 375 males and 362 female patients, with a ratio of 1.04:1 (χ² = 20.047, P = 0.045). However, it can be found that the gender ratio in 2015 is particularly low. After excluding data in 2015, there is no statistically significant (χ² = 6.88, P = 0.737). The number of deaths in males and females is 44 and 38, with mortality rates of 11.7% and 10.5% for males and females, respectively (χ² = 0.285, P = 0.594) (S3A Fig). The average age SFTS patients was 64.79 years, ranging from 2 to 93 (S3B Fig). The majority cases aged 60–69 years accounting for 31.3% (231/737). The patients are mainly farmers, accounting for 73.3%, followed by unemployees, accounting for 16.0% (S3 Table).

### Global and local autocorrelation analysis

The Moran’I index of annual SFTS incidence rate in Zhejiang Province is different from 2011 to 2022 (S4 Table). There is spatial clustering in each year (P < 0.05) except for 2011, 2012, and 2014 (P > 0.05).

**Fig 3:**
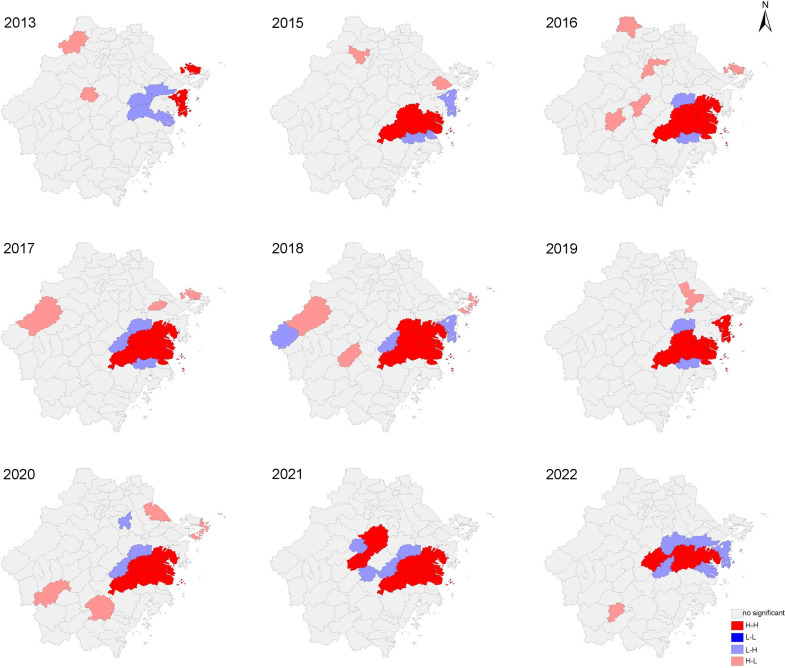
LISA cluster map of local autocorrelation analysis of SFTS in Zhejiang Province from 2011 to 2022. The base layer of the map is from DataV.GeoAtlas, a website providing standard maps that are approved for public use and are in compliance with the national standards for map representation(http://datav.aliyun.com/portal/school/atlas/area_selector#&lat=31.769817845138945&lng=104.29901249999999&zoom=4).

There was local spatial autocorrelation in SFTS in Zhejiang, and the most explicit part was the H-H cluster. The H-H gathering areas were mainly in the hilly terrain of the east coast of Zhejiang province, and the range of the area is expanding with time. In 2013, H-H gathering areas were distributed in Zhoushan, and from 2015 to 2020, they were mainly distributed in Linhai, Tiantai County, Sanmen County, Xianju County, Jiaojiang District, Ninghai County and Xiangshan County of Taizhou. In 2021–2022, the gathering areas of Taizhou decreased and shifted to Jinhua and Shaoxing (Fig 3).

### Risk factors for SFTS

Potentially risk factors, ecoclimatic, land cover, and social variables were analyzed to find the relative contribution to the occurrence of SFTS using BRT models (Table 1). We selected variables based on their correlation with the SFTS case distribution, the availability of data, and the explanatory power of the model. For example, environmental factors, such as vegetation cover and land-use type, directly affect tick distribution and human activity patterns, thereby influencing the spread of SFTS. In addition, climatic conditions such as temperature and precipitation have also been shown to be associated with the prevalence of SFTS. The results revealed that all three factors contributed to the occurrence of the disease. The BRT mean weights of mean temperature of wettest quarter was highest (18.51%), followed by annual precipitation (9.29%), and the percentage coverage of grassland (9.06%). Variables with mean weights≥ 5% were considered as significant contributors to the occurrence of human infections. Overall, there were 7 factors have significant relative contribution to the occurrence of SFTS, including the mean temperature of wettest quarter, annual precipitation etc., and these key factors affecting the spread of SFTS can help to develop targeted prevention strategies, such as strengthening personal protection measures.

**Table 1:** Results of the boosted regression trees applied to the occurrence of SFTS data.

Variables	Relative contribution	
Mean (%)	Sd
Mean temperature of wettest quarter (°C)	18.51	3.22
Annual precipitation (mm)	9.29	1.53
The percentage coverage of grassland (%)	9.06	0.75
Human population density (per square kilometer)	8.03	1.85
The percentage coverage of forest (%)	7.59	0.94
Temperature seasonality	5.67	0.76
Mean temperature of driest quarter (°C)	5.18	1.48
Precipitation of driest quarter (mm)	4.19	1.58
Mean temperature of coldest quarter (°C)	4.08	0.63
Precipitation of coldest quarter (mm)	4.08	1.10
The percentage coverage of water (%)	4.06	0.57
Mean diurnal range (Mean of monthly (max temp-min temp)) (°C)	3.07	0.84
The percentage coverage of cropland (%)	2.85	0.40
Precipitation of warmest quarter (mm)	2.76	0.51
The percentage coverage of impervious (%)	2.61	0.40
Mean temperature of warmest quarter (°C)	2.19	0.18
The percentage coverage of shrub (%)	2.17	0.15
The percentage coverage of barren (%)	2.03	0.01

Note: Variables with mean weights≥ 5% were considered as significant contributors to the occurrence of human infections.

**Fig 4:**
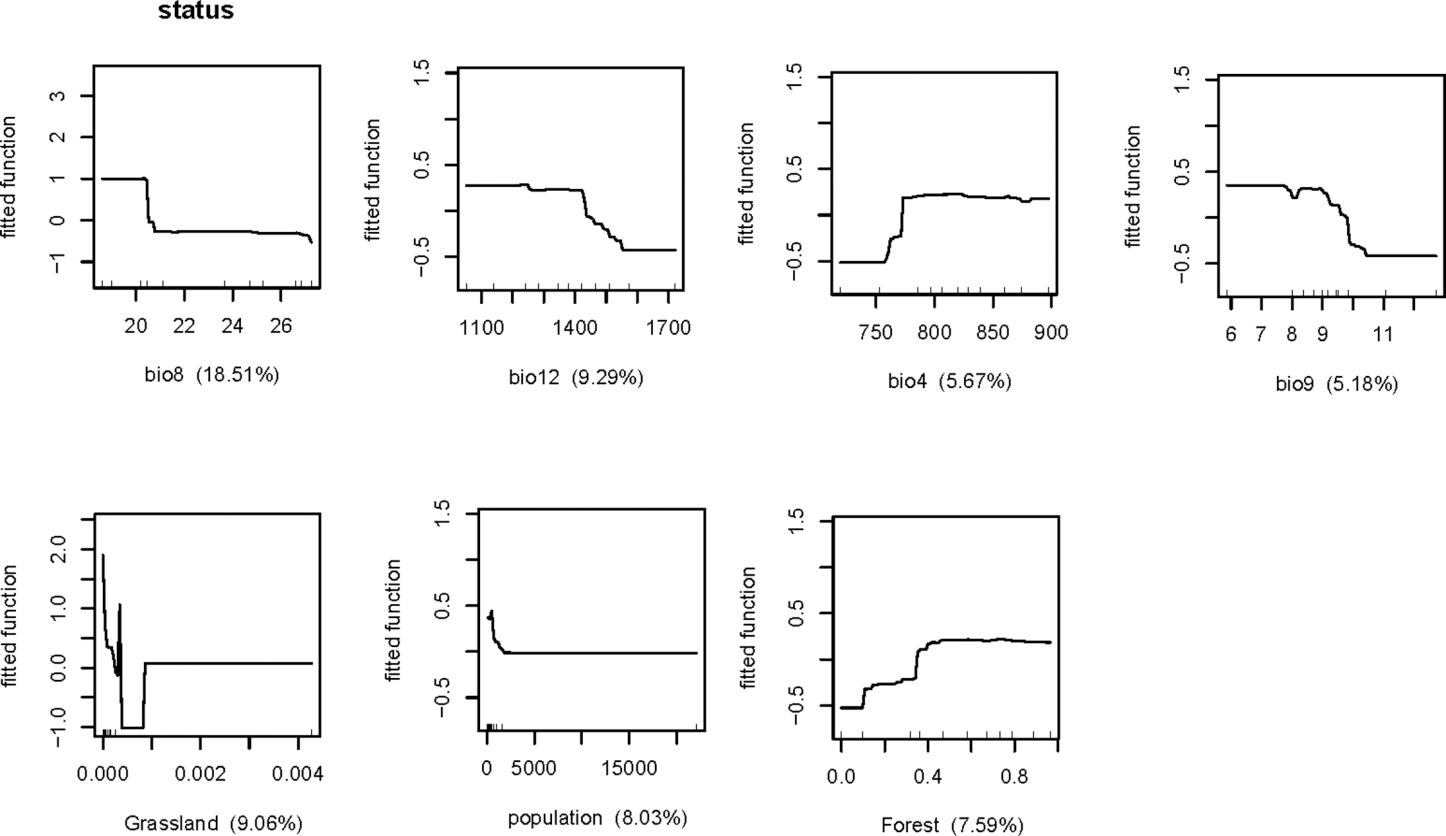
Relationship between risk factors and SFTS risk. BIO04, temperature seasonality; BIO08, mean temperature of wettest quarter; BIO09, mean temperature of driest quarter; BIO12, annual precipitation; grassland, the percentage coverage of grassland; population, human population density; forest, the percentage coverage of forest.

The fitted function plot based on the BRT model revealed that the occurrence of SFTS decreased with mean temperature of wettest quarter, mean temperature of driest quarter, annual precipitation, and human population density, whereas temperature seasonality and the percentage coverage of forest manifested an opposite trend (Fig 4). Specifically, when mean temperature of wettest quarter reached 20°C and human population density reached 1000 person per km2, the risk dropped sharply. The influence of the percentage coverage of grassland demonstrated a totally different pattern. When the percent below 0.001, the risk declined and then rose, and then remained stable. The percentage coverage of grassland and human population density were largely clustered at the low end of the presented range with a single value much higher than these, so the results should be interpreted with caution.

**Fig 5:**
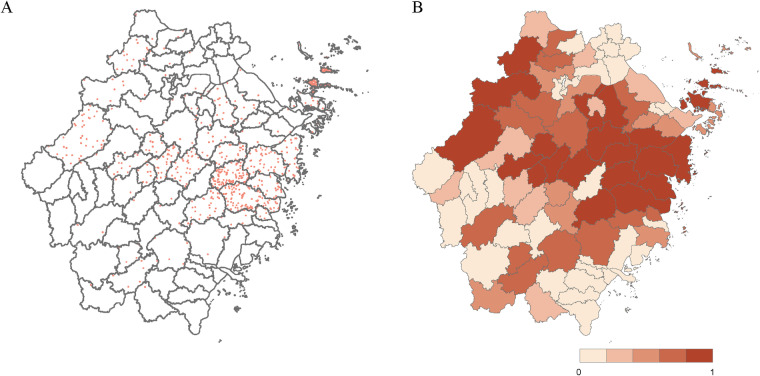
The reported distributions (A) and model-predicted occurrence probability (B) of SFTS cases at the county level in Zhejiang province from 2011-2022. The probabilities derived are at the county level. The base layer of the map is from DataV.GeoAtlas, a website providing standard maps that are approved for public use and are in compliance with the national standards for map representation. (http://datav.aliyun.com/portal/school/atlas/area_selector#&lat=31.769817845138945&lng=104.29901249999999&zoom=4).

The model-predicted risk areas resembled the current reporting regions (Fig 5), clustering mainly in the hilly terrain of the east coast of Zhejiang province, especially in Daishan County (0.986), Linhai city (0.972), and Tiantai County (0.971) with higher probability. The dots in the figure is a result of the visualization technique used in ArcMap 10.8. Notably, there are more areas predicted than reported. Those regions predicted possible cases but did not report were concentrated in western, southern, and northern Zhejiang, including Quzhou, Lishui, Wenzhou, and Jiaxing City. To evaluate the discriminatory power, the receiver-operating characteristic (ROC) curve and area under the curve (AUC) was calculated for the BRT model. The ecological modeling results showed highly accurate predictions, with the average testing AUC ranging from 0.9786 to 0.9993 (S4 Fig).

## Discussion

In this study, the spatial and temporal characteristics of SFTS were explored in Zhejiang Province from 2011 to 2022, with a total of 737 cases. The results suggested that SFTS cases showed an obvious seasonality, as most cases reported during April-August. There was spatial cluster of SFTS cases, indicating that the disease was not randomly distributed in Zhejiang Province. Mean temperature of wettest quarter, annual precipitation, the percentage coverage of grassland, human population density, the percentage coverage of forest, temperature seasonality, and mean temperature of driest quarter are significant factors which influence the distribution of SFTS.

This study found that the annual average incidence of SFTS was 0.099/100000 in Zhejiang province, which was lower than the national incidence (0.11/100000) [24] and the neighboring Anhui province in the same period (0.58/100000) [25], but higher than that in Jiangsu province (0.059/100000) [26]. The incidence showed an increasing trend year by year and a peak (0.16/100000) was observed in 2022. The case fatality rate was decreasing year by year, but remaining in a high level. Meanwhile, it was also observed that the number of counties reporting cases was increasing year by year in Zhejiang province. These results may be related to multiple factors, such as an increase in the distribution and density of ticks in the natural environment, an increase in the frequency of public outdoor activities, an increase in health concerns, and a significant improvement in the ability of medical institutions to detect and diagnose diseases [5,27]. In addition, the public health surveillance systems have allowed more cases to be detected and reported timely. However, SFTS patients are generally more severe and some may not receive timely diagnosis and treatment at the early stage of the disease, the mortality rate remains high. This also suggests the need to further strengthen the prevention and control of SFTS, improve public health awareness, and optimize the allocation of medical resources in order to further reduce the mortality of SFTS.

The incidence of SFTS had high seasonality, with the epidemic peak month from April to August, which is consistent with previous studies [28]. The seasonal distribution of ticks may be related to the life cycle of ticks, the dynamics of ticks and the duration of human outdoor activities. H. Longicornis is the major media of SFTS and its distribution is closely related to meteorological factors [29].The BRT model identified temperature and precipitation as significant environmental factors affecting the risk of SFTS (Fig 4). This result is highly consistent with the biological characteristics of H. longicornis and existing research. The temperature around 17 °C was conducive to ticks’ growth and reproduction, resulting in an increasing density of ticks [30]. It is possible that temperatures above 17 °C make the tick habitat less hospitable, leading to a decrease in SFTS. Precipitation maintains vegetation cover and soil moisture, providing a suitable microenvironment for ticks, but higher precipitation may lead to habitat inundation, which is not conducive to their survival [30], which explains negative relationship between precipitation and SFTS risk in Fig 4. According to a survey [31], H. Longicornis is the dominant tick species of Zhejiang Province and H. Longicornis was dominant in Zhoushan, Taizhou, and Jinhua, which is consistent with the geographical distribution of reported cases. Tick metabolism, growth rate, and fecundity are enhanced in a warm and humid environment, whereas these activities are slowed down in a cool and dry climate [15]. In addition, outdoor activities such as tea-picking and lawn-mowing were carried out by farmers during this period. In addition, people dressed less in summer and autumn, thus may increase the chance of contact with host animals carrying SFTSV.

SFTS cases are mainly concentrated in hilly and mountainous areas in Zhejiang Province such as Daishan County, Zhoushan City, Tiantai County, Taizhou City, and Linhai City. Daishan County is the area where the first SFTS case has been reported in Zhejiang Province, with approximately 20.2% of cases occurring here. This area belonging to the island hilly area with high vegetation coverage, and is mainly dominated by shrubs, which is suitable for the survival and reproduction of various vector organisms and rodents. H. longicornis, an important vector of SFTS transmission, mainly inhabits these shrub areas [23,32]. According to historical monitoring data, the tick density in Daishan County is at a relatively high level, which undoubtedly increases the risk of SFTS transmission in the region [28]. Therefore, the incidence of SFTS in Daishan County may be closely related to the unique geographical environment of the local area. Meanwhile, this study found that the number of cases in Zhoushan, where Daishan County is located, has declined significantly since 2016. This change may be the result of a series of comprehensive community-based interventions implemented in Daishan County since 2016. These measures included health education and risk communication to raise public awareness and awareness of SFTS and to clean up litter and weeds to reduce vector breeding grounds and regular use of chemical insecticides to directly reduce the number and activity of vector organisms.

The high incidence of SFTS cases in Zhejiang province occurred among middle-aged and elderly people, and most cases are farmers, which may be related to exposure to ticks. It is closely associated with the current social structure and changes in the rural labor force. With the young and middle-aged people in rural areas working in cities, the elderly remain in hometown and have become the main force of outdoor work such as farming and tea picking. They work in fields or tea plantations, which are the ideal breeding places for ticks [33]. In addition, elderly people are more susceptible to infection due to declines in immune function and comorbidities [34]. These findings suggested that we should pay special attention to the health status of the elderly in the public health prevention and control work, and provide necessary protective measures and health guidance.

The results on ecological drivers for SFTS diseases in human bear some similarity with previous studies. For example, a study based on surveillance data of laboratory-confirmed SFTS cases in China found that elevation, close-canopy woodland, shrubland, and precipitation in the driest quarter were important drivers for SFTS from 2010-2018 [35]. Another study also reported temperature seasonality (relative contribution: 16.89%), mean temperature wettest quarter (10.07%), and annual temperature range (9.46%) [23]. And precipitation, mean temperature, and relative humidity also showed a non-linear relationship with SFTS cases [36]. Similarly, in the present study, among the ecoclimatic factors, the most influential factor for SFTS is mean temperature of wettest quarter followed by annual precipitation. The wettest quarter often overlaps with the summer season in Zhejiang province. Consequently, the summer season is a key to the occurrence of SFTS. The present study also indicated the important role of human population density. The incidence of SFTS decreased with increasing population density. This may be because areas with higher population densities have better health resources and greater capacity for disease surveillance and diagnosis, allowing cases to be detected and treated earlier. It could also be related to less tick habitat present in higher density locations. These higher density locations could contain more urban land covers and less vegetation, reducing suitable locations for tick populations and reducing the potential for human-tick encounters.

By showing the predicted probability of each county, we can more intuitively identify high-risk areas, which has important implications for public health decision-making and resource allocation. Notably, the predicted distribution of cases was wider than reported, especially in Quzhou, Lishui, Wenzhou, and Jiaxing City. The topography of Zhejiang province is complex, and the whole terrain descends from southwest to northeast, and the southwest is mainly mountainous [37], where the environmental conditions are suitable for the survival of ticks, which might be responsible for the expanded areas of prediction. Additionally, human activity might play a role. High-frequency human movement in tick-infested areas, such as for tourism or business purposes in regions with suitable tick habitats, was not incorporated into the model. Further research is needed to explore the role of these factors such in the model. Each county should take measures tailored to its specific situation, such as improving disease surveillance systems and strengthening diagnostic capacity.

The advantage of this study was that we constructed BRT model with data on SFTS incidence, ecoclimatic factors, land cover, and human population density from 2011 to 2022 in Zhejiang province. Those maps demonstrate the probability of occurrence of SFTS and help managers and policymakers understand the geographic risk distribution of the disease and identify high-risk areas. Public health officials can better target resources, plan interventions, and monitor disease trends. In addition, these maps can assist decision makers in assessing the effectiveness of existing control measures and adjusting strategies according to changes in risk distribution. There are also several limitations. First, the incidence and case fatality rate of SFTS might be underestimated due to the lack of effective diagnostic methods in remote medical facilities and the lack of disease surveillance systems or some patients with adverse clinical progression were discharged for economic reasons [5]. Our data may be less constrained by this, as residents in less developed counties and remote areas in Zhejiang also have timely access to medical resources (https://fzggw.zj.gov.cn/art/2021/7/28/art_1229123366_2313094.html). Second, the BRT model was constructed based on annual incidence at the county level. The relatively large space and time scale may lead to ecological fallacy. For example, if a county has a high incidence rate, we may mistakenly infer that all individuals in that county are at a high risk of infection, ignoring differences between individuals. Third, there may be more factors associated with SFTS that have not been explored. For instance, tick species and density could affect SFTS incidence. However, data on tick species and density were not collected.

## Conclusions

In summary, SFTS is highly seasonal with the peaking season spanning from April to August, also showing inter-regional differences. The spatiotemporal characteristics were presented and ecological and social drivers of the emerging SFTS were explored in Zhejiang province from 2011-2022. As a result, SFTS reflects a sensitive response to ecoclimatic factors, land cover, and human population density. Nevertheless, the precise effect of these factors on SFTS the during the complex processes warrants further study. According to the predicted results of the model, there may be many potential cases that have not been discovered, indicating the need to strengthen monitoring of SFTS and SFTSV. The findings of this study can provide important information to public health officials to help them better understand the geographical risk distribution of SFTS. Active surveillance of SFTS is recommended in counties with ecological suitability for the tick vector in the future, especially those high-risk areas having no SFTS cases reported yet.

## Supporting information

S1 Table Descriptive statistics (mean and range) of all covariates in counties (N = 737) reporting SFTS cases in Zhejiang province. (DOCX)

S2 Table Descriptive statistics of significant covariates in BRT models based on case-control data set. (DOCX)

S3 Table The number and composition ratio of cases of SFTS in Zhejiang Province across different occupations from 2011 to 2022. (DOCX)

S4 Table Global spatial autocorrelation analysis of SFTS in Zhejiang Province from 2011 to 2022. (DOCX)

S1 Data The Epidemiological data of STFS cases. (XLSX)

S2 Data Case-control data including ecoclimatic, land cover, and social factors used for BRT analysis. (XLSX)

S3 Data The predicted SFTS incidence probability per county by the BRT model. (XLSX)

S1 Fig Location of provinces, cities, and counties mentioned in the article. (A) A map of the spatial location of Zhejiang province in China and other provinces mentioned in our article; (B) A map of the spatial location of 11 cities in Zhejiang province; (C) A map of the spatial location of counties in Zhejiang province. These maps were created by Rnaturalearth package with R software (version 4.3.3, http://www.r-project.org/).
(TIF)

S2 Fig Monthly distribution of SFTS cases in Zhejiang Province from 2011 to 2022. (TIF)

S3 Fig A, The number of male and female cases of SFTS in Zhejiang Province each year from 2011 to 2022; B, The number of male and female cases in different age groups and case fatality rates of SFTS in Zhejiang Province from 2011 to 2022. (TIF)

S4 Fig ROC curves of the predicted risk of SFTS presence/absence. ROC curves for BRT models: the grey lines are the ROC curve for each repeat, and the black and red lines indicate the average ROC curves of 50 repeats based on the bootstrapping procedure for the train set and test set.
(TIF)
