# Supplementary material for: Epidemiological characteristics and risk distribution prediction of severe fever with thrombocytopenia syndrome in Zhejiang Province, China
Source: PLoS Negl Trop Dis. 2025 Apr 25;19(4):e0013066. doi: 10.1371/journal.pntd.0013066 (PMC12054904; doi:10.1371/journal.pntd.0013066)
Supplement: S1 Table — (DOCX) [file pntd.0013066.s001.docx]

S1 Table. Descriptive statistics (mean and range) of all covariates in counties (N = 737) reporting SFTS cases in Zhejiang province.

| Category | Variable | Description | Mean (Minimum-Maximum) |
| --- | --- | --- | --- |
| Ecoclimatic | BIO01 | Annual mean temperature (℃) | 16.83 (14.27-17.92) |
|  | BIO02 | Mean diurnal range (Mean of monthly (max temp-min temp)) (℃) | 7.32 (5.80-8.96) |
|  | BIO03 | Isothermality (BIO02/BIO07) (*100) | 24.70 (21.83-29.98) |
|  | BIO04* | Temperature seasonality (standard deviation*100) | 815.38(724.29-897.66) |
|  | BIO05 | Max temperature of warmest month (℃) | 31.51 (29.30-33.70) |
|  | BIO06 | Min temperature of coldest month (℃) | 1.97 (-1.30-3.80) |
|  | BIO07 | Annual range of temperature (BIO05-BIO06) (℃) | 29.54 (26.30-32.30) |
|  | BIO08 | Mean temperature of wettest quarter (℃) | 21.85 (18.60-27.25) |
|  | BIO09 | Mean temperature of driest quarter (℃) | 9.05 (5.87-12.57) |
|  | BIO10 | Mean temperature of warmest quarter (℃) | 26.61 (24.10-27.93) |
|  | BIO11 | Mean temperature of coldest quarter (℃) | 6.67 (3.83-8.22) |
|  | BIO12 | Annual precipitation (mm) | 1327.40 (1140-1722) |
|  | BIO13 | Precipitation of wettest month (mm) | 198.64 (152-308) |
|  | BIO14 | Precipitation of driest month (mm) | 40.83 (33.00-52.00) |
|  | BIO15 | Precipitation seasonality (Coefficient of variation) | 45.00(38.41-59.01) |
|  | BIO16 | Precipitation of wettest quarter (mm) | 491.99 (383-758) |
|  | BIO17 | Precipitation of driest quarter (mm) | 153.53 (118-185) |
|  | BIO18 | Precipitation of warmest quarter (mm) | 459.62 (362-634) |
|  | BIO19 | Precipitation of coldest quarter (mm) | 169.53 (141-209) |
| Land cover | Cropland | The percentage coverage of cropland (%) | 23.33 (2.34-77.06) |
|  | Forest | The percentage coverage of forest (%) | 58.01 (0.03-96.06) |
|  | Grassland | The percentage coverage of grassland (%) | 0.01 (0-0.09) |
|  | Water | The percentage coverage of water (%) | 4.23 (0.04-18.77) |
|  | Barren | The percentage coverage of barren (%) | <0.01 (0-0.08) |
|  | Impervious | The percentage coverage of impervious (%) | 8.82 (0.5-34.67) |
|  | Shrub | The percentage coverage of shrub (%) | <0.01 (0-0.09) |
|  | Wetland | The percentage coverage of wetland (%) | <0.01 (0-0) |
|  | Snow and Ice | The percentage coverage of snow or ice (%) | <0.01 (0-0) |
| Social | human population density | The number of human population / total area (per square kilometer) | 503.60 (56.00-  5559.75) |

*BIO04, temperature seasonality, this is a commonly used indicator in ecological studies to describe seasonal fluctuations in climate variables.
