# Supplementary material for: Epidemiological characteristics and risk distribution prediction of severe fever with thrombocytopenia syndrome in Zhejiang Province, China
Source: PLoS Negl Trop Dis. 2025 Apr 25;19(4):e0013066. doi: 10.1371/journal.pntd.0013066 (PMC12054904; doi:10.1371/journal.pntd.0013066)
Supplement: S2 Table — (DOCX) [file pntd.0013066.s002.docx]

S2 Table. Descriptive statistics of significant covariates in BRT models based on case-control data set.

| Variable | Description | Mean (Minimum-Maximum) |
| --- | --- | --- |
| BIO08 | Mean temperature of wettest quarter (℃) | 22.95 (18.60-27.25) |
| BIO12 | Annual precipitation (mm) | 1369.34 (1051.00-1722.00) |
| Barren | The percentage coverage of grassland (%) | <0.01 (0-0) |
| human population density | Human population density (per square kilometer) | 1029.35 (54.32-22098.25) |
| Forest | The percentage coverage of forest (%) | 0.52 (0-0.98) |
| BIO04 | Temperature seasonality | 818.81 (719.08-897.66) |
| BIO09 | Mean temperature of driest quarter (℃) | 9.26 (5.87-12.72) |

* Significant covariates are those with a relative contribution greater than 5% estimated from the BRT model.
