# Supplementary material for: Epidemiological characteristics and risk distribution prediction of severe fever with thrombocytopenia syndrome in Zhejiang Province, China
Source: PLoS Negl Trop Dis. 2025 Apr 25;19(4):e0013066. doi: 10.1371/journal.pntd.0013066 (PMC12054904; doi:10.1371/journal.pntd.0013066)
Supplement: S3 Table — (DOCX) [file pntd.0013066.s003.docx]

S3 Table. The number and composition ratio of cases of SFTS in Zhejiang Province across different occupations from 2011 to 2022.

| Occupation | Number of cases | Ratio (%) |
| --- | --- | --- |
| Farmer | 540 | 73.3 |
| Unemployee | 118 | 16.0 |
| Others | 79 | 10.7 |
