# Supplementary material for: Epidemiological characteristics and risk distribution prediction of severe fever with thrombocytopenia syndrome in Zhejiang Province, China
Source: PLoS Negl Trop Dis. 2025 Apr 25;19(4):e0013066. doi: 10.1371/journal.pntd.0013066 (PMC12054904; doi:10.1371/journal.pntd.0013066)
Supplement: S4 Table — (DOCX) [file pntd.0013066.s004.docx]

S4 Table. Global spatial autocorrelation analysis of SFTS in Zhejiang Province from 2011 to 2022.

| Year | Moran'I | *Z* value | *P* value | Aggregation |
| --- | --- | --- | --- | --- |
| 2011 | -0.038 | -0.448 | 0.269 | No |
| 2012 | 0.060 | 1.794 | 0.071 | No |
| 2013 | 0.491 | 8.436 | 0.002 | Yes |
| 2014 | -0.018 | -0.150 | 0.405 | No |
| 2015 | 0.288 | 5.890 | 0.002 | Yes |
| 2016 | 0.380 | 6.391 | 0.004 | Yes |
| 2017 | 0.444 | 7.236 | 0.001 | Yes |
| 2018 | 0.239 | 3.890 | 0.007 | Yes |
| 2019 | 0.278 | 4.632 | 0.003 | Yes |
| 2020 | 0.240 | 4.041 | 0.005 | Yes |
| 2021 | 0.252 | 4.104 | 0.004 | Yes |
| 2022 | 0.248 | 3.968 | 0.007 | Yes |
