# Supplementary figures and images for: Epidemiological characteristics and risk distribution prediction of severe fever with thrombocytopenia syndrome in Zhejiang Province, China
Source: PLoS Negl Trop Dis. 2025 Apr 25;19(4):e0013066. doi: 10.1371/journal.pntd.0013066 (PMC12054904; doi:10.1371/journal.pntd.0013066)

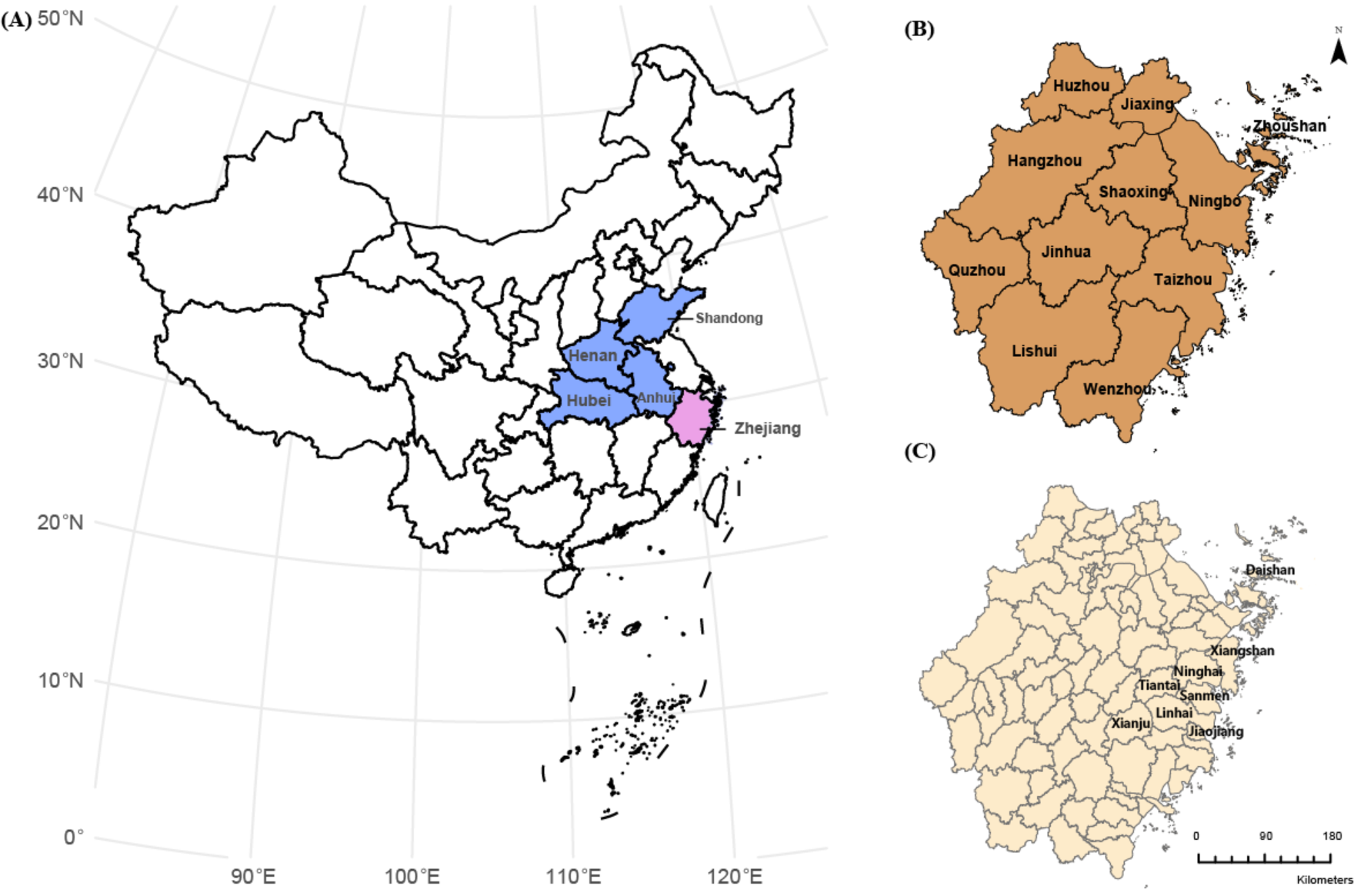

Supplement: S1 Fig — (A) A map of the spatial location of Zhejiang province in China and other provinces mentioned in our article; (B) A map of the spatial location of 11 cities in Zhejiang province; (C) A map of the spatial location of counties in Zhejiang province. These maps were created by Rnaturalearth package with R software (version 4.3.3, http://www.r-project.org/). (TIF) [file pntd.0013066.s008.tif]

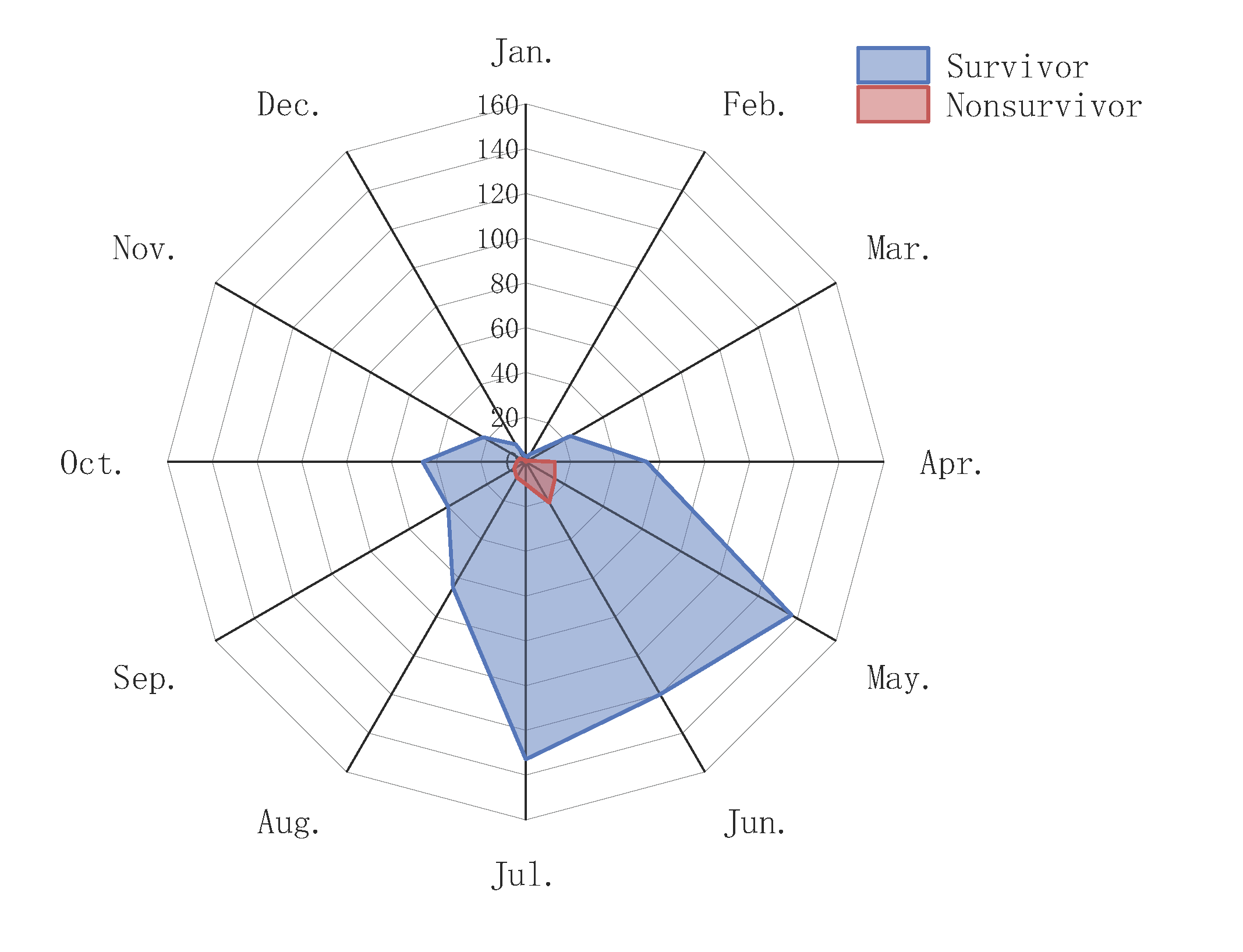

Supplement: S2 Fig — (TIF) [file pntd.0013066.s009.tif]

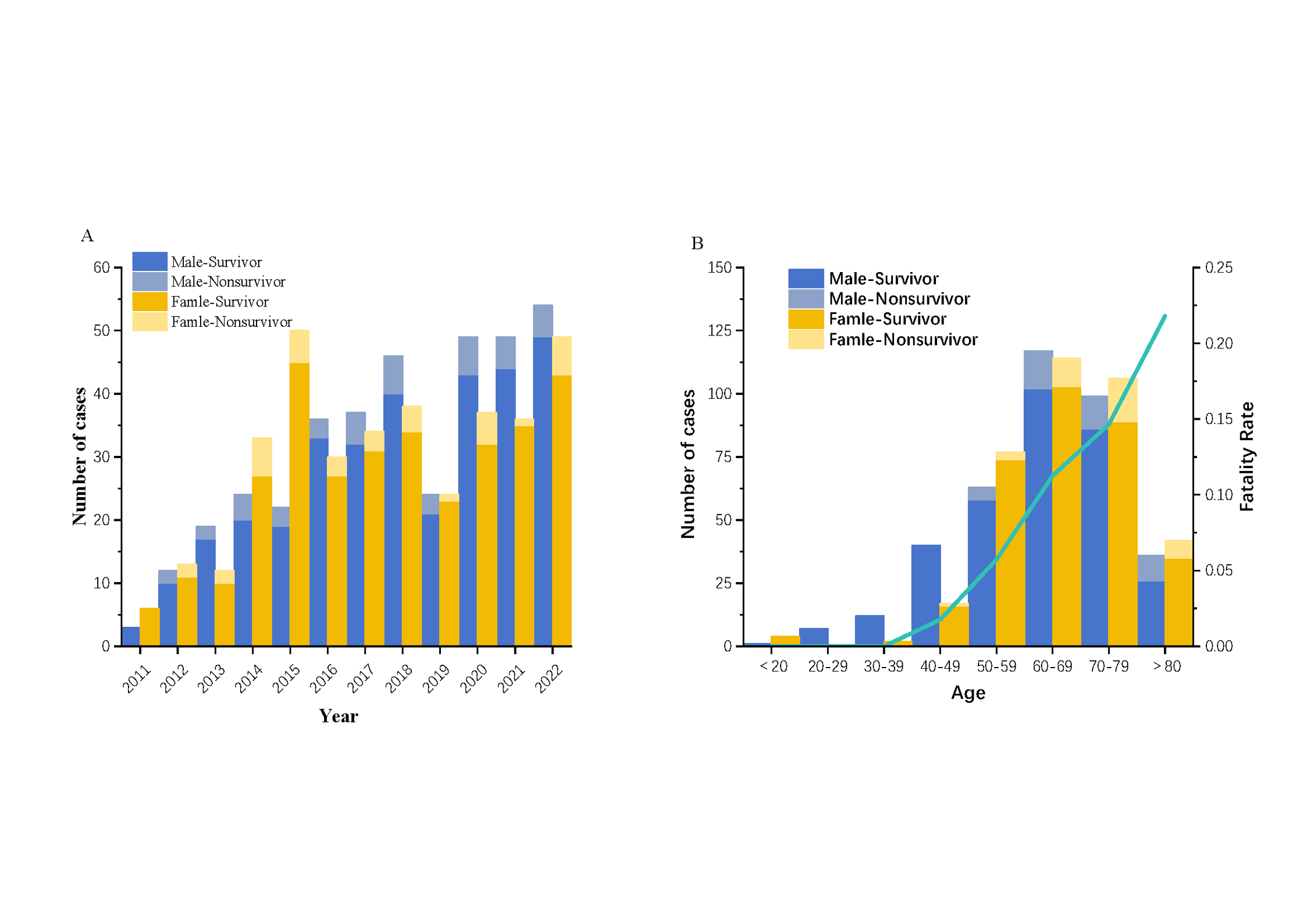

Supplement: S3 Fig — (TIF) [file pntd.0013066.s010.tif]

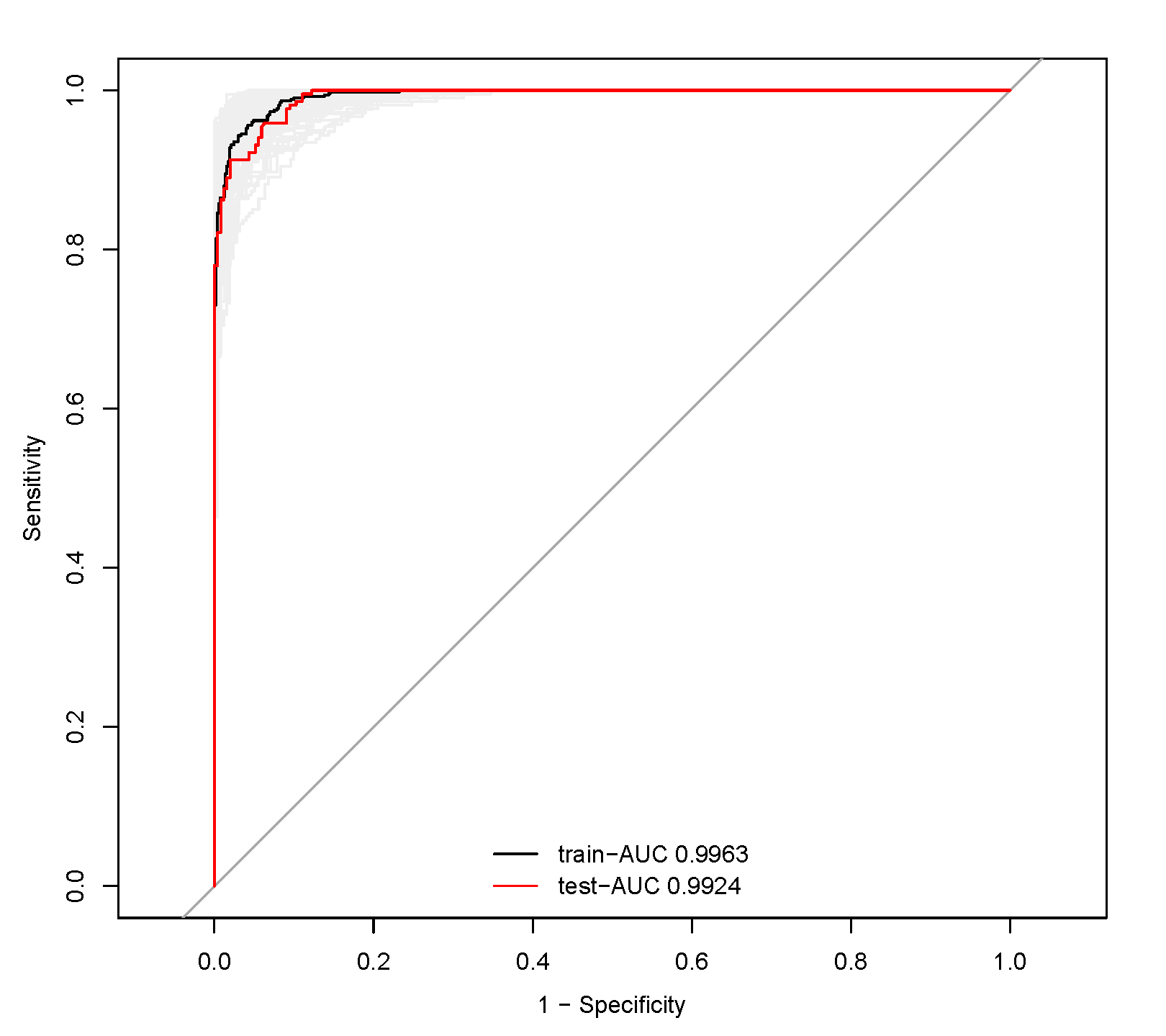

Supplement: S4 Fig — ROC curves for BRT models: the grey lines are the ROC curve for each repeat, and the black and red lines indicate the average ROC curves of 50 repeats based on the bootstrapping procedure for the train set and test set. (TIF) [file pntd.0013066.s011.tif]
